# Real-Time Monitoring of Cardiac Output Using Non-Invasive Impedance Cardiography in Dogs: A Pilot Study on Heartworm Extraction and Gastric Decompression

**DOI:** 10.3390/vetsci12050478

**Published:** 2025-05-15

**Authors:** Daesik Kim, Seol-Gi Park, Min Su Kim

**Affiliations:** 1 Veterinary Emergency Medicine, Department of Veterinary Clinical Science, Research Institute for Veterinary Science, College of Veterinary Medicine, Seoul National University, Seoul 08826, Republic of Korea; daxic88@snu.ac.kr; 2 Incheon Sky Animal Hospital, Incheon 21555, Republic of Korea; roronanah@gmail.com

**Keywords:** gastric dilatation, heartworm, cardiac output, non-invasive monitoring, dog, interventional extraction

## Abstract

Background: Gastric dilatation (GD) and heartworm disease (HW) can significantly impact cardiovascular function, necessitating timely intervention. This study evaluated hemodynamic changes in these conditions using impedance cardiography (ICG), a non-invasive technique for real-time monitoring. Methods: Cardiac parameters were measured using ICG in healthy controls, HW, and GD groups at baseline, during intervention, and post-treatment. Statistical analyses involved repeated measures ANOVA with post hoc comparisons, and relative changes (%) quantified hemodynamic improvements. Results: A significant increase in cardiac index (CI) occurred post-treatment in both HW (+14.71%, *p* = 0.0102) and GD (+28.53%, *p* = 0.0336) groups. Cardiac output (CO) exhibited an increasing trend, though without significance (*p* > 0.05). Mean arterial pressure (MAP) remained stable, although the GD group exhibited an upward trend post decompression (*p* = 0.1213). Conclusions: Heartworm extraction and gastric decompression induced measurable cardiovascular improvements, with distinct recovery patterns. Non-invasive monitoring effectively captured cardiovascular changes, suggesting its utility as an adjunctive tool in clinical assessment.

## Simple Summary

Non-invasive monitoring tools can significantly improve veterinary care for critically ill animals. This study investigated impedance cardiography (ICG) as a method to monitor heart function in dogs with two serious conditions: gastric dilatation and heartworm disease. We examined how key cardiovascular parameters changed before, during, and after standard treatments for these conditions. Our findings revealed that both heartworm extraction and gastric decompression led to measurable improvements in heart function, with cardiac index increasing by 14.71% and 28.53%, respectively. These results demonstrate that ICG can effectively detect hemodynamic changes without invasive procedures, offering veterinarians a practical tool for monitoring patient response to treatment. This approach could improve clinical decision-making and potentially lead to better outcomes for dogs with these critical conditions.

## 1. Introduction

Accurate and timely monitoring of cardiac output (CO) is essential in managing patients with critical cardiovascular conditions. CO, defined as the volume of blood ejected by the heart per minute, is a fundamental indicator of cardiac function and overall hemodynamic status [1,2]. Traditional methods for measuring CO, such as thermodilution, transesophageal echocardiography (TEE), arterial pressure waveform analysis (APWA), and CT/MRI-based evaluation, have specific advantages and limitations. Thermodilution is highly accurate and considered the gold standard [3,4]; however, it is invasive, technically demanding, and carries risks such as infection and bleeding [5]. TEE provides detailed cardiac imaging but is invasive and may induce discomfort or complications in patients with esophageal disorders [6]. APWA allows continuous real-time monitoring but can exhibit significant inaccuracies under specific hemodynamic conditions [7]. While CT and MRI deliver precise three-dimensional cardiac assessments, their high costs and inability to perform real-time monitoring limit practical use in emergency or acute care settings [8,9,10]. These limitations underscore the need for a non-invasive, reliable, and real-time CO monitoring technique, particularly in critical and rapidly evolving clinical situations.

Impedance cardiography (ICG) has emerged as a promising non-invasive modality for continuous cardiac output (CO) monitoring. It provides real-time hemodynamic data by detecting thoracic electrical impedance changes associated with each cardiac cycle, without requiring catheter insertion. Its simplicity and rapid deployment make it highly attractive for a variety of clinical scenarios [4,11]. Although its absolute accuracy can be influenced by factors such as thoracic conformation or fluid accumulation, ICG has consistently demonstrated reliable sensitivity in detecting directional changes in CO over time [12,13,14]. Direct comparisons between ICG and the gold standard thermodilution (TD) method have shown moderate agreement, with electrical cardiometry (a form of ICG) achieving a bias of −0.14 L/min and concordance rates exceeding 85% for trend detection [4,7]. Compared to other non-invasive methods like echocardiography and arterial pressure waveform analysis, ICG offers continuous real-time monitoring without operator dependency or the need for arterial catheterization [12,15,16,17]. Furthermore, a comprehensive evaluation of multiple CO monitoring techniques under hemodynamic stress confirmed that, while ICG’s absolute accuracy remains modest, its favorable trend-tracking ability is clinically valuable [7]. It was originally developed for human medicine, but now it is increasingly used in veterinary medicine [16,17].

Heartworm (HW) disease significantly impacts cardiac output (CO) through increased pulmonary artery pressure, right ventricular overload, and tricuspid valve dysfunction, leading to impaired forward flow [18,19]. Similarly, gastric dilatation (GD) leads to CO reduction due to increased intra-abdominal pressure and impaired venous return [4,20]. Additionally, vagally mediated bradycardia further suppresses CO [21,22,23]. Although heartworm disease and gastric dilatation differ in their pathophysiological mechanisms—with the former involving progressive pulmonary hypertension and the latter characterized by acute mechanical compression of major vessels—both result in compromised cardiac output and require rapid intervention expected to elicit abrupt hemodynamic shifts. This study aimed to evaluate the feasibility and utility of non-invasive ICG for real-time CO monitoring in patients diagnosed with HW and GD.

By assessing CO variations during parasite removal in HW and decompression procedures in GD, this study sought to determine whether ICG can provide consistent trend-tracking data to support clinical decision-making in acute care settings, despite potential variations in absolute accuracy under different physiological conditions.

The aim of this study was to evaluate the feasibility and utility of non-invasive impedance cardiography (ICG) for real-time cardiac output monitoring in dogs with heartworm disease (HW) and gastric dilatation (GD). We hypothesized that ICG would reliably detect relative hemodynamic changes associated with therapeutic interventions (heartworm extraction and gastric decompression), thereby supporting its potential use as a clinical monitoring tool. This study specifically evaluated cardiac output (CO) along with associated parameters, including cardiac index (CI) and mean arterial pressure (MAP), to characterize hemodynamic changes before, during, and after intervention. Through this investigation, we explored whether ICG could provide a practical, efficient, and sensitive method for tracking cardiac function, particularly in conditions characterized by rapid fluctuations in CO. These findings may provide a basis for future studies and broader clinical evaluation of ICG in veterinary emergencies and critical care settings.

## 2. Material and Methods

This study was conducted in compliance with ethical guidelines for animal research and was approved by the Institutional Animal Care and Use Committee (IACUC) of Seoul National University (Approval No. SNU-200226-7-2, SNU-240115-9-1). A total of 16 dogs were included and categorized into three groups: six healthy controls, six HW cases, and four GD cases. The healthy control group was used to establish reference values for CO and mean arterial pressure (MAP), while the HW and GD groups underwent interventional procedures with ICG monitoring before and after treatment. The control group consisted of six healthy adult Beagle dogs (age range: 2–4 years; body weight: 5.5–10.0 kg). All animals were purpose-bred and had not been previously enrolled in any experimental procedures. Prior to inclusion in this study, each dog underwent a comprehensive health screening that included a thorough physical examination, complete blood count, serum biochemistry profile, thoracic radiography, and abdominal ultrasonography to rule out any underlying disease. Only dogs with no clinical abnormalities and normal laboratory and imaging findings were included as controls. All animals were housed in a controlled environment with standardized feeding and care protocols throughout the study period.

**Figure 1:**
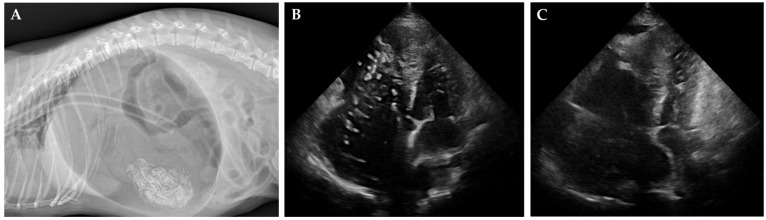
**Radiographic and echocardiographic findings in cases of gastric dilatation and heartworm disease.** (**A**) Lateral radiograph of a dog with severe gastric dilatation, showing a markedly distended, gas-filled stomach. An orogastric tube for decompression is visible. (**B**,**C**) Right parasternal short-axis echocardiographic images before (**B**) and after (**C**) heartworm removal. Heartworms appear as short, segmented, parallel echogenic structures (**B**), which are no longer visible post-extraction (**C**).

The HW and GD groups consisted of client-owned dogs that presented for therapeutic intervention (Figure 1). For the HW group, dogs included various breeds such as Poodle, Yorkshire Terrier mix, Pit Bull Terrier, and mixed-breed dogs (age range: 2–12 years; body weight: 3.72–46.0 kg). Patient selection was based on the American Heartworm Society (AHS) guideline criteria, specifically targeting dogs for whom surgical heartworm extraction was indicated due to significant worm burden or right atrial involvement as determined by echocardiography. All HW cases presented with moderate to severe heartworm disease, characterized by multiple adult worms visualized within the pulmonary arteries and/or right heart chambers. Mild clinical signs such as intermittent coughing, mild exercise intolerance, and mild ascites were observed in some cases. While none of the HW dogs in this study had developed caval syndrome at the time of intervention, we recognize the importance of including such cases to fully capture the severe end of the disease spectrum. All dogs exhibited mild to moderate pulmonary hypertension on echocardiography without evidence of severe right ventricular dysfunction or overt right-sided heart failure.

For the GD group, dogs included Dachshund, Samoyed, and Welsh Corgi breeds (age range: 1–8 years; body weight: 6.62–23.5 kg). Diagnosis of acute gastric dilatation was made based on physical examination, a detailed history, and confirmation with abdominal radiographs. Clinical signs included abdominal distension, non-productive retching, and varying degrees of cardiovascular compromise. Based on clinical assessment at presentation, one dog was classified as having severe cardiovascular compromise (poor pulse quality and prolonged capillary refill time), two dogs exhibited moderate compromise, and one showed mild cardiovascular effects. Emergency stabilization, including orogastric decompression and/or surgical intervention, was promptly performed. In all GD cases, ICG monitoring was conducted after initial stabilization to minimize the effects of acute hemodynamic instability on measurement reliability.

Informed consent for participation, including the use of non-invasive impedance cardiography (ICG) monitoring during therapeutic procedures, was obtained from all owners prior to enrollment.

### 2.1. Impedance Cardiography Setup

**Figure 2:**
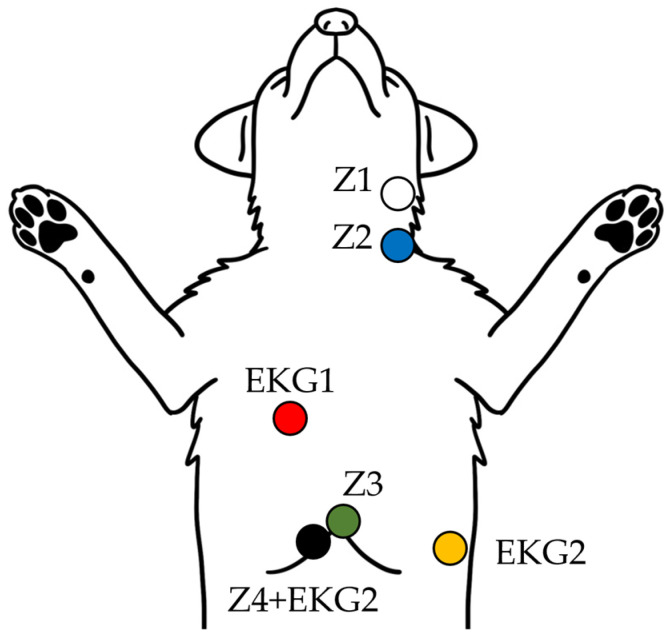
**Schematic diagram of ICG and EKG electrode placement in dogs.** The illustration shows the standardized surface electrode placement used to acquire thoracic impedance (Z1–Z4) and ECG (EKG1–2) signals for non-invasive cardiac output monitoring in dogs positioned in dorsal recumbency.

Impedance cardiography (ICG) is a non-invasive technique for continuous cardiac output (CO) and stroke volume (SV) monitoring. In this study, the PhysioFlow^®^ system (version 1.0.7 RC9.15 SVV Edition Lab1 Enduro, Manatec Biomedical, Bristol, PA, USA) was used to obtain real-time hemodynamic data. This device utilizes Signal-Morphology Impedance Cardiography (SM-ICG™) technology, which analyzes the shape of the impedance waveform rather than relying on baseline thoracic impedance (Z0), allowing for more robust measurements even under variable clinical conditions. ICG operates by transmitting a low-magnitude, high-frequency alternating current through the thorax via surface electrodes. Thoracic impedance changes (ΔZ) during the cardiac cycle are detected and differentiated (dZ/dt), corresponding to aortic blood flow velocity. Six electrodes were applied according to a standardized configuration [12]: two were placed dorsally at the level of the 4th and 6th cervical vertebrae near the left jugular vein, one on the right side of the sternum at the 5th intercostal space, one on the left lateral thorax near the 13th rib, and two just below the xiphoid process (Figure 2). To optimize signal acquisition, electrodes were attached using clip-type leads with conductive gel after clipping and cleaning the skin with alcohol. Placement accuracy was maintained within ±3 cm of anatomical landmarks, and each setup was verified by two operators to ensure consistency. The system was calibrated according to manufacturer specifications before each monitoring session. Only data with a signal quality ≥80% were included in the analysis, and measurements contaminated by motion artifacts or electrical interference were excluded. ICG measurement intervals were set at 5 s, and 15 repeated values were acquired per phase for each dog, allowing for both assessment of immediate changes and calculation of averaged values. For the GD group, CO and other hemodynamic parameters were measured in three distinct phases: before decompression (pre-decompression), immediately after decompression (post-decompression), and during the stabilization phase (post-stabilization). These measurements evaluated the hemodynamic instability caused by elevated intra-abdominal pressure and its subsequent relief. For the HW group, CO monitoring was conducted also in three phases: prior to the extraction procedure (pre-extraction), during HW extraction (extraction phase), and during the recovery phase after extraction (post-stabilization).

This consistent approach provided a comprehensive and accurate assessment of the hemodynamic effects during interventions in both the GD and HW groups. Both CO and CI values were derived from impedance measurements, with CI calculated as CO normalized to body surface area (BSA) and expressed in liters per minute per square meter (L/min/m^2^).

### 2.2. Experimental Protocol

Prior to enrollment, all dogs underwent a physical examination and baseline diagnostic screening, including complete blood count, serum biochemistry, and thoracic radiography or ultrasonography when indicated, to ensure the absence of significant comorbidities unrelated to heartworm disease or gastric dilatation. GD cases were retrospectively confirmed to be free of other underlying conditions following full clinical workup during hospitalization. All dogs (NC, HW, and GD groups) received butorphanol (0.2–0.4 mg/kg IV) as premedication. To reduce procedural side effects, each dog was medicated starting from 3 days prior to the procedure and continuing up to the day of extraction. This pre-treatment was intended to minimize potential perioperative complications during heartworm removal. The medications administered included prednisolone (0.5 mg/kg PO BID), doxycycline monohydrate (5 mg/kg PO BID), silymarin (20 mg/kg PO SID), famotidine (0.5 mg/kg PO BID), clopidogrel hydrogen sulfate (2 mg/kg PO SID), cefaclor (20 mg/kg PO BID), and furosemide (1 mg/kg PO SID). In the NC and HW groups, general anesthesia was induced with alfaxalone (1–4 mg/kg IV, titrated to effect) and maintained with isoflurane (1–4%) following tracheal intubation. In the GD group, sedation was performed using butorphanol alone, with one dog undergoing endotracheal intubation and general anesthesia for surgical correction, and the remaining three managed without intubation during gastric decompression.

For the healthy control group (NC, *n* = 6), a standardized anesthesia protocol was implemented to evaluate ICG measurement stability in the absence of pathological conditions or interventional procedures. Following initial physical examination and confirmation of health status, these animals underwent general anesthesia maintained for a total duration of one hour. During this period, no specific interventions were performed, allowing assessment of hemodynamic parameter stability under controlled conditions.

CO and MAP measurements were obtained at three distinct time points: (1) stabilization phase (15–20 min after anesthesia induction, once cardiovascular parameters had stabilized), (2) mid-anesthesia phase (approximately 30 min into anesthesia), and (3) late anesthesia phase (approximately 50–60 min into anesthesia). This protocol was designed specifically to evaluate the consistency and reliability of ICG measurements over time in subjects with presumed stable hemodynamic status, thereby establishing both reference ranges and the trending capability of the monitoring system.

In the HW group, impedance cardiography (ICG) monitoring was performed across three defined phases: (1) pre-extraction phase: baseline cardiac output (CO) and mean arterial pressure (MAP) were recorded after anesthesia induction, jugular venotomy, and placement of the heartworm extraction device under fluoroscopic guidance. Measurements were obtained during a period of cardiovascular stability before initiating heartworm removal; (2) during extraction phase: continuous real-time monitoring of CO and MAP was conducted throughout the mechanical heartworm extraction process. Data acquisition was performed at 5 s intervals, and motion artifacts were excluded based on signal quality. This phase spanned from the start of worm retrieval to the completion of extraction; (3) post-extraction stabilization phase: following the completion of heartworm extraction, CO and MAP measurements continued during a 10–15 min stabilization period. Again, 15 measurements were collected per patient to assess cardiovascular recovery and detect any delayed hemodynamic changes. This structured protocol allowed for comprehensive evaluation of hemodynamic trends associated with heartworm removal. Post-extraction stabilization measurements were taken after completion of the heartworm extraction procedure to evaluate recovery and detect any residual hemodynamic instability. The heartworm extraction procedure was performed using a basket retrieval device (S & G Biotech Inc., Seoul, Republic of Korea) under fluoroscopic guidance.

In the GD group, ICG monitoring was also conducted in three phases: pre-decompression, post-decompression, and post-stabilization. Pre-decompression measurements established baseline hemodynamic parameters before the intervention. Post-decompression measurements were recorded immediately after the procedure to assess immediate CO changes. Post-stabilization monitoring continued until hemodynamic parameters stabilized. GD was performed via orogastric intubation, followed by surgical correction when necessary. In one GD case, hemodynamic measurements were performed after initial fluid therapy and successful orogastric decompression, during a period of clinical stabilization. All measurements were completed within a 15 min using a standardized three-phase protocol (pre-decompression, during decompression, and immediate post-decompression). Approximately 30 min after the final measurement, the dog developed recurrent gastric distension and was diagnosed with gastric dilatation-volvulus (GDV), requiring emergency surgical intervention. The procedure included exploratory laparotomy, gastric de-rotation, viability assessment of abdominal organs, and prophylactic gastropexy.

### 2.3. Statistical Analysis

Statistical analyses were conducted using GraphPad Prism version 10.3.0 (GraphPad Software, San Diego, CA, USA). The primary focus of this study was to evaluate within-group hemodynamic changes over time during therapeutic interventions, rather than to perform direct comparisons between different disease groups or against controls. Normality of the data distributions was assessed using the Shapiro–Wilk test. Since the assumption of normality was satisfied (*p* > 0.05), repeated measures ANOVA (RM-ANOVA) was applied for within-group comparisons among three time points (Pre, Post, and Stabilization). When the assumption of sphericity was violated, the Geisser–Greenhouse correction was applied. Tukey’s post hoc test was used for multiple pairwise comparisons. Multiplicity-adjusted *p*-values were reported, and statistical significance was considered at *p* < 0.05. Additionally, 95% confidence intervals (CIs) were calculated where appropriate to enhance the interpretation of clinical significance. Non-parametric analyses were conducted using the Friedman test to validate findings, followed by Wilcoxon signed-rank tests for pairwise comparisons. To account for inter-individual variability, changes in cardiac index (CI) were expressed as relative change percentages, calculated using the following formula:

(1) $$Relative\;Change\;(\%)=\frac{Post-treatment\;Value-Pre-treatment\;Value}{Pre-treatment\;Value}\times 100$$

A *p*-value of <0.05 was considered statistically significant.

## 3. Results

### 3.1. Post-Procedure Outcomes and Recovery

All NC dogs successfully underwent the procedures without complications and recovered uneventfully from anesthesia without any adverse effects. In the HW group, post-procedural echocardiographic examination confirmed a reduction in heartworm burden, allowing the dogs to proceed with the standard heartworm treatment protocol [24]. Representative radiographic and echocardiographic findings for diseased dogs (HW and GD groups) are shown (Figure 1). In the GD group, all dogs were hospitalized for continuous monitoring after decompression, with no recurrence of GD observed. The average hospitalization period was 5.75 ± 0.63 days, and at the three-month follow-up examination, no abnormalities were detected, confirming successful long-term recovery. There were no significant differences in age, weight, or gender distribution between the three groups.

### 3.2. Hemodynamic Changes Following Intervention

**Figure 3:**
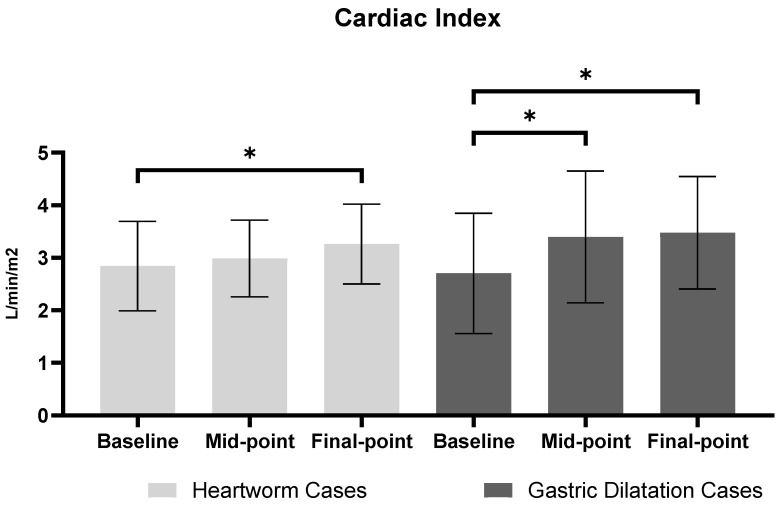
**Cardiac Index comparison between heartworm and gastric dilatation cases across three time points.** Values are presented as mean ± SEM (standard error of mean). For Heartworm cases, time points represent: baseline (anesthesia stabilization), Mid-point (during extraction), and Final point (after extraction). For gastric dilatation cases, time points represent: baseline (pre-decompression), mid-point (post-decompression), and final-point (stabilization). Asterisks (*) indicate statistically significant differences between time points (*p* < 0.05).

To evaluate the effects of treatment on hemodynamic parameters, cardiac output (CO), cardiac index (CI), and mean arterial pressure (MAP) were measured at baseline, during the procedure, and at the final phase. Hemodynamic data expressed as mean ± SEM with 95% confidence intervals (CIs) are summarized (Table 1). In addition to parametric analyses, non-parametric analyses using the Friedman test and Wilcoxon signed-rank tests were conducted to validate the robustness of the findings. The results confirmed significant improvements in cardiac output (CO) and cardiac index (CI) after intervention, consistent with the parametric analysis outcomes (Table 2). Furthermore, time-course changes in cardiac index (CI), expressed as percentage changes relative to baseline, and their corresponding statistical significance are summarized (Table 3). In the normal control (NC) group, stable anesthesia and recovery were observed throughout the procedure. Hemodynamic parameters, including CO, CI, and MAP, remained stable without significant fluctuations (*p* > 0.05), indicating well-maintained physiological stability under anesthesia and post-procedural recovery. In the heartworm disease (HW) group, a significant improvement in cardiac index (CI) was observed after heartworm removal compared to baseline. Post-procedural echocardiographic examination confirmed a reduction in heartworm burden, allowing the dogs to proceed with the standard heartworm treatment protocol [16]. However, changes in cardiac output (CO) and mean arterial pressure (MAP) did not reach statistical significance. In the gastric dilatation (GD) group, cardiac output (CO) and cardiac index (CI) increased significantly after decompression, indicating substantial improvement in cardiac performance. Although mean arterial pressure (MAP) also tended to increase, the change was not statistically significant. The time-course changes in cardiac index (CI) within each disease group, illustrating significant improvements after intervention, are shown (Figure 3).

## 4. Discussion

This study evaluated the feasibility of non-invasive real-time CO monitoring in dogs with HW and GD using ICG. By establishing the baseline CO and MAP values from healthy dogs, this study enabled a comparative assessment of hemodynamic changes before and after therapeutic interventions in HW and GD cases. The incorporation of a normal control (NC) group in this study design served dual methodological purposes. Primarily, it established reference ranges for ICG-derived hemodynamic parameters in clinically healthy canines under standardized conditions. These normative data are particularly valuable given the paucity of established reference intervals for ICG measurements in veterinary medicine. The observed stability in hemodynamic parameters among control subjects (CI: 2.706 ± 0.101 L/min/m^2^ at baseline with nonsignificant variations of 0.40% and 2.12% at subsequent time points) provides critical context for interpreting pathophysiological alterations in disease states.

Our methodological approach prioritized within-group hemodynamic changes in response to therapeutic interventions. The comparable baseline CI values observed across the three groups merit careful consideration from multiple perspectives. In chronic heartworm disease, physiologic compensation—such as increased heart rate and myocardial contractility—may maintain near-normal resting cardiac output despite underlying pulmonary vascular compromise. Similarly, during the early stages of gastric dilatation, cardiovascular dysfunction may be present without substantial reductions in CI at the time of measurement, potentially due to initial resuscitative measures administered prior to ICG monitoring. Additionally, the intrinsic characteristics of impedance cardiography warrant consideration regarding the potential overestimation of CI in disease groups. Anatomical alterations in heartworm disease or gastric dilatation (e.g., increased pulmonary vascular resistance and elevated intra-abdominal pressure) might affect impedance measurements, yielding CI values that appear higher than the actual hemodynamic status. These considerations further support the utility of ICG as a trend-monitoring tool rather than a method for absolute hemodynamic quantification.

Furthermore, the consistent measurements obtained across multiple time points in the NC group (stabilization, sedation, and anesthesia stabilization phases) demonstrate the reliability and reproducibility of ICG technology for serial hemodynamic assessments, which is commonly referred to as “trending ability” in hemodynamic monitoring literature [4,12,25]. This technical validation aspect is essential when evaluating a monitoring modality intended for continuous assessment during dynamic clinical scenarios.

The hemodynamic stability observed in the NC group stands in marked contrast to the significant temporal variations detected in both intervention groups. The quantifiable improvements in CI following heartworm extraction (+14.71%, *p* = 0.0102) and gastric decompression (+33.81%, *p* = 0.0336) represent statistically significant hemodynamic responses to therapeutic interventions rather than measurement artifacts. This differentiation between pathophysiological stability and intervention-induced change substantiates the clinical utility of ICG for real-time assessment of cardiovascular responses to treatment.

These findings suggest that ICG not only provides absolute hemodynamic values but also reliably tracks directional changes in cardiac performance over time. This trending capability may prove particularly valuable in emergency and critical care settings, where rapid the assessment of treatment efficacy and early detection of hemodynamic deterioration can significantly impact patient outcomes.

In both heartworm extraction and gastric decompression procedures, systemic blood pressure alone may be insufficient to determine the optimal endpoint of intervention. In our study, several HW cases exhibited a measurable increase in cardiac output following parasite removal yet mean arterial pressure (MAP) remained suboptimal or fluctuated—a finding consistent with prior experimental studies involving heartworm-associated hemodynamic changes [17,26]. Similarly, in GD cases, a significant improvement in cardiac index (28.53%, *p* = 0.0336) following decompression was not proportionally reflected in MAP values. This dissociation underscores the limitations of relying solely on arterial pressure for clinical decision-making. The use of impedance cardiography (ICG) allowed continuous tracking of cardiac performance trends throughout both procedures, providing critical real-time data beyond conventional monitoring parameters. These additional insights enabled more comprehensive evaluation of the physiological response to treatment and supported clinicians in determining appropriate endpoints for intervention. Our findings suggest that ICG may serve as a valuable adjunctive monitoring tool in emergency and critical care settings, especially for conditions such as heartworm disease and gastric dilatation, where traditional vital signs may inadequately reflect the true hemodynamic status.

The PhysioFlow^®^ system addresses several technical challenges inherent to impedance cardiography. Importantly, the system’s HD-Z™ filtering technology mitigates the potential interference between respiratory and cardiac cycles by analyzing impedance waveform morphology rather than relying on baseline thoracic impedance (Z0). The system samples at 250 Hz, capturing multiple cardiac cycles within each 5 s measurement interval, which enables reliable trend monitoring despite varying respiratory patterns during anesthesia. This signal processing approach effectively isolates cardiac-related impedance changes from respiratory artifacts, maintaining measurement consistency even during mechanical ventilation. While factors such as electrode placement, thoracic conformation, and tissue characteristics may influence absolute values, the system’s focus on waveform analysis rather than absolute impedance values helps maintain reliable trend tracking. This capability was evident in our study, where consistent hemodynamic response patterns were observed across different clinical scenarios, supporting ICG’s utility as a trend-monitoring tool during critical interventions.

Non-invasive methods for measuring cardiac output (CO) are becoming increasingly relevant, particularly in small animal research where minimizing procedural risks is a priority [1,25,27,28]. Among these, impedance cardiography (ICG) has gained attention for its ability to track stroke volume (SV) and CO in real-time by detecting thoracic electrical conductivity changes throughout the cardiac cycle [29]. This technique has proven valuable in human neonatal and pediatric patients, where continuous, non-invasive monitoring is essential [30]. While its clinical application in veterinary medicine is still under investigation, ongoing studies suggest its potential for accurate and practical CO assessment [4,9,11,16,31,32,33,34].

In heartworm (HW) disease, CO measurement using pulmonary artery catheterization is often impractical, as the invasive nature of the procedure can interfere with parasite extraction. Additionally, HW-related cardiovascular alterations, such as pulmonary hypertension and right ventricular dysfunction, necessitate continuous CO monitoring to evaluate hemodynamic responses. Similarly, gastric dilatation (GD) presents unique challenges, as decompression induces sudden shifts in venous return and CO. In GD cases, rapid intervention is critical, making the use of invasive monitoring devices impractical due to the time required for insertion and stabilization. In contrast, ICG can be set up within 10 min, with minimal disruption to ongoing procedures [4]. Furthermore, its non-invasive nature ensures minimal interference with other critical interventions, allowing for seamless integration into emergency treatment protocols. Therefore, in this study, ICG was selected as a non-invasive tool capable of providing real-time CO monitoring in both HW and GD cases, allowing for continuous assessment of cardiovascular stability during critical interventions.

In the GD group, a moderate CO decrease was observed before decompression, likely due to impaired venous return caused by increased intra-abdominal pressure. Following decompression, ICG detected a rapid CO recovery, supporting the effectiveness of decompression in restoring hemodynamic stability. This immediate improvement in CO can be attributed not only to the relief of intra-abdominal pressure but also to the concurrent administration of intravenous fluid therapy. The increased venous return from aggressive fluid resuscitation likely contributed to the rapid hemodynamic restoration observed post-decompression [35,36].

Previous studies have demonstrated ICG’s potential for non-invasive CO monitoring in human and veterinary medicine [13], yet its application in HW and GD cases remains underexplored. Traditional CO measurement methods, such as Doppler echocardiography and thermodilution, provide accurate assessments but are often invasive, operator-dependent, or impractical for continuous monitoring [8,15]. Unlike these techniques, ICG offers real-time CO measurement with minimal procedural burden, making it more suitable for acute and emergency settings [11].

In this study, the ICG was able to detect transient CO fluctuations during HW extraction, a feature that is typically undetected by static measurement techniques. Our finding suggests that ICG may be used to complement existing hemodynamic monitoring tools by providing continuous real-time data, particularly in conditions with dynamic cardiovascular changes.

This study has limitations that should be addressed in future research. The small sample size (GD: 4, HW: 6) limits the generalizability of the findings. Future studies with larger and more diverse populations are essential to validate the broader applicability of ICG. Additionally, reliable data acquisition from ICG requires skillful electrode placement and stable signal quality. Improper placement or signal interference during emergencies may lead to inaccurate readings, which could adversely affect clinical outcomes. Further advancements in electrode design and signal-processing algorithms are necessary to enhance data stability and accuracy.

Additionally, ICG’s reliance on thoracic impedance and ECG-based calculations may pose challenges in patients with arrhythmias, such as premature ventricular contractions (VPCs), potentially affecting data accuracy [22,37]. To improve the clinical applicability of ICG, the development of advanced algorithms to provide reliable measurements under arrhythmic conditions is worth considering.

Importantly, while ICG provides valuable hemodynamic data, it should be viewed as an adjunctive tool only. Clinical expertise is indispensable in interpreting ICG data in the context of a patient’s overall condition, history, and other monitoring parameters. While ICG is a powerful adjunct to guide therapeutic interventions, effective decision-making requires integrating all available clinical information, supported by a skilled medical team [37,38]. In this study, transient arrhythmic events were observed in some GD cases, particularly during the initial stabilization phase. However, these episodes were short-lived, and stable sinus rhythm was restored prior to ICG measurement. All reported hemodynamic data were obtained during periods of rhythm stability, minimizing the influence of arrhythmias on the interpretation of CI and MAP trends.

Future research should investigate the efficacy of ICG in larger and more diverse populations across various clinical conditions. By addressing these challenges, ICG has the potential to become a cornerstone technology for real-time hemodynamic monitoring, particularly for dynamic cardiovascular conditions.

## 5. Conclusions

This study explored the potential utility of non-invasive impedance cardiography (ICG) for real-time CO monitoring in canine HW and GD cases. By providing immediate trend-based hemodynamic data, ICG demonstrated promise as a monitoring tool that could support clinical decision-making during interventions characterized by acute CO fluctuations. The consistent detection of hemodynamic improvement following therapeutic interventions suggests ICG may offer valuable insights into cardiovascular responses, though larger studies are needed to confirm these preliminary findings.

ICG’s non-invasive application, rapid implementation, and concurrent monitoring of CO alongside standard MAP measurements provides more comprehensive hemodynamic assessment than conventional monitoring alone. This combined monitoring approach shows potential clinical utility in emergencies and critical care settings where complete cardiovascular data can inform intervention strategies. This pilot study suggests that integrating ICG with traditional monitoring parameters may offer clinicians additional insights into cardiovascular status during critical interventions in canine patients. With further validation through expanded clinical trials, this combined hemodynamic assessment approach could enhance monitoring capabilities in veterinary emergencies and routine care.

## Figures and Tables

**Table 1:** Summary of patient characteristics.

Group	Parameter	Baseline	Mid-Point	Final-Point
Normal Control (*n* = 6)	CO (L/min)	1.217 ± 0.053 (95% CI: 1.113–1.321)	1.212 ± 0.049 (95% CI: 1.116–1.308)	1.244 ± 0.054 (95% CI: 1.138–1.350)
	CI (L/min/m2)	2.706 ± 0.101 (95% CI: 2.508–2.904)	2.689 ± 0.090 (95% CI: 2.513–2.865)	2.760 ± 0.098 (95% CI: 2.568–2.952)
	MAP (mmHg)	86.689 ± 0.860 (95% CI: 85.003–88.375)	85.478 ± 0.710 (95% CI: 84.086–86.870)	85.967 ± 0.659 (95% CI: 84.675–87.259)
Heartworm Disease (HW) (*n* = 6)	CO (L/min)	1.563 ± 0.529 (95% CI: 0.526–2.600)	1.660 ± 0.527 (95% CI: 0.627–2.693)	1.786 ± 0.581 (95% CI: 0.647–2.925)
	CI (L/min/m2)	2.844 ± 0.088 (95% CI: 2.636–3.052)	2.989 ± 0.080 (95% CI: 2.678–3.114)	3.263 ± 0.086 * (95% CI: 2.652–3.068)
	MAP (mmHg)	76.067 ± 0.861 (95% CI: 74.379–77.755)	76.483 ± 0.851 (95% CI: 74.815–78.151)	76.517 ± 0.685 (95% CI: 75.174–77.860)
Gastric Dilatation (GD) (*n* = 4)	CO (L/min)	1.775 ± 0.108 (95% CI: 1.544–2.006)	2.220 ± 0.132 (95% CI: 1.961–2.479)	2.288 ± 0.131 (95% CI: 2.031–2.545)
	CI (L/min/m2)	2.706 ± 0.137 (95% CI: 2.437–2.975)	3.397 ± 0.148 * (95% CI: 3.107–3.687)	3.500 ± 0.147 * (95% CI: 3.212–3.788)
	MAP (mmHg)	56.567 ± 1.212 (95% CI: 54.191–58.943)	69.700 ± 0.790 (95% CI: 68.152–71.248)	70.967 ± 0.686 (95% CI: 69.622–72.312)

Values are presented as mean ± SEM (standard error of mean), with 95% confidence intervals (CIs) provided separately below each value. Time points: normal control group—stabilization, process, end phase; heartworm disease (HW) group—pre-extraction, during extraction, post-extraction; gastric dilatation (GD) group—pre-decompression, post-decompression, stabilization. * *p* < 0.05 compared to baseline within the same group. CO = cardiac output; CI = cardiac index; MAP = mean arterial pressure.

**Table 2:** Non-parametric analysis results (Friedman test and Wilcoxon signed-rank test).

Group	Parameter	Friedman Test *p*-Value	Significant Pairwise Comparisons (Wilcoxon Test)	Notes
Heartworm Disease (HW) (*n* = 6)	CO (L/min)	*p* < 0.05	Pre vs. During (*p* < 0.05), Pre vs. Post (*p* < 0.05), During vs. Post (n.s.)	Increase from Pre to Post
	CI (L/min/m2)	*p* < 0.05	Pre vs. During (*p* < 0.05), Pre vs. Post (*p* < 0.05), During vs. Post (n.s.)	Increase from Pre to Post
	MAP (mmHg)	0.84	Not applicable	No significant difference
Gastric Dilatation (GD) (*n* = 4)	CO (L/min)	*p* < 0.01	Pre vs. Post (*p* < 0.01), Pre vs. Stabilization (*p* < 0.01), Post vs. Stabilization (n.s.)	Increase after decompression
	CI (L/min/m2)	*p* < 0.01	Pre vs. Post (*p* < 0.01), Pre vs. Stabilization (*p* < 0.01), Post vs. Stabilization (n.s.)	Increase after decompression
	MAP (mmHg)	*p* < 0.01	Pre vs. Post (*p* < 0.01), Pre vs. Stabilization (*p* < 0.01), Post vs. Stabilization (n.s.)	Increase after decompression

Summary of the non-parametric analysis results using the Friedman test and post hoc Wilcoxon signed-rank tests. Significant differences between specific time points are indicated. Abbreviations: HW: heartworm disease group; GD: gastric dilatation group; CO: cardiac output; CI: cardiac index; MAP: mean arterial pressure; n.s.: not significant.

**Table 3:** Time-course changes in cardiac index and statistical significance.

Group	Baseline CI	Mid-Point Change (%)	*p*-Value (Mid-Point)	Final-Point Change (%)	*p*-Value (Final-Point)
**Normal Control** **(*n* = 6)**	2.706 ± 0.101	0.40	>0.05	2.12	>0.05
**Heartworm Disease** **(*n* = 6)**	2.844 ± 0.088	5.09	>0.05	14.71	0.0102 *
**Gastric Dilatation** **(*n* = 4)**	2.706 ± 0.137	28.26	0.0184 *	33.81	0.0336 *

CI values are presented as mean ± SEM (L/min/m^2^). Time points: normal control—stabilization→process→end phase; HW group—pre-extraction→during extraction→post-extraction; GD group—pre-decompression→post-decompression→stabilization. Relative change (%) = (post-treatment value—pre-treatment value)/pre-treatment value × 100. (*) Bold values indicate statistically significant changes (*p* < 0.05).

## Data Availability

The data presented in this study are available in this manuscript.
